# Follicular Biochemical Characterization and Fatty Acid Metabolic Signatures of Follicles During Ovulation Process Reveal the Potential Mechanism for Ovarian Cyst Formation in Sows

**DOI:** 10.3390/metabo15070421

**Published:** 2025-06-20

**Authors:** Jingyuan Liang, Yanfei Deng, Song Fu, Juanru Cheng, Ruimen Zhang, Deshun Shi, Yu Pan, Sufang Yang

**Affiliations:** 1 Guangxi Key Laboratory of Animal Breeding and Disease Control, College of Animal Science and Technology, Guangxi University, Nanning 530004, China; 2018401006@st.gxu.edu.cn (J.L.); yanfei-dun@163.com (Y.D.); s3023503655@163.com (S.F.); 2118401014@st.gxu.edu.cn (J.C.); 20230099@gxu.edu.cn (R.Z.); ardsshi@gxu.edu.cn (D.S.); 2 Chongqing Academy of Animal Sciences, Chongqing 402460, China; 3 Guangxi international Zhuang Medical Hospital, Guangxi University of Chinese Medicine, Nanning 530000, China

**Keywords:** ovulation, fatty acids, follicular fluid, ovarian cyst, linoleic acid metabolism, linoleic acid, arachidonic acid

## Abstract

**Background/Objectives**: As a well-known source of energy from feed, the significance of fatty acids in regulating the reproductive potential of livestock has received attention in recent years, especially follicular development. Moreover, successful ovulation is a process that is crucial for reproduction and fertility in domestic animals. Therefore, it is important to reveal the signatures of fatty acids in follicular fluid during mammalian ovulation, and this provides a possible method to prevent the occurrence of ovarian cysts in domestic animals. **Methods**: Pre-ovulatory follicles (*n* = 6) and peri-ovulatory follicles (*n* = 6) during normal ovulation, as well as cystic follicles (*n* = 6) in ovulation-deficient ovarian cyst were isolated and characterized, while follicular fluid was collected for targeted fatty acid metabolomics detection and analysis. **Results**: We have illustrated the anatomical and biochemical characterization of pre-ovulatory, peri-ovulatory, and cystic follicles. Subsequently, we identified changes in 51 fatty acids profiles in the follicular fluid. The highest proportion of fatty acids in the follicular fluid at three different ovulation stages is polyunsaturated fatty acids, among which the abnormality of the linoleic acid metabolism pathway was involved in ovulation defects in cystic follicles. Remarkably, we found that linoleic acid was significantly increased while arachidonic acid was significantly decreased in cystic follicles. **Conclusions**: Polyunsaturated fatty acids play a significant role in the follicular ovulation stage of sows. Among them, linoleic acid and arachidonic acid are closely related to the ovulation defects of cystic follicles, which suggests that identifying changes in important metabolic signatures may give us a better understanding of the pathogenesis of ovarian cyst.

## 1. Introduction

The deficiencies and excesses of nutrients can influence reproductive performance and cause reproductive disorders [1]. Ovarian cysts are one of the common infertility diseases in female animals, resulting in a complex result of disturbances in follicular development and ovulation. Statistically, the incidence of ovarian cysts has been reported to be 5–18% in fertile women [2], 2.8–4.2% in buffaloes [3], 5.6–18.8% in cows [4], 3–16% in dogs [5], and 2.5–40% in sows [6]. Currently, it is well-known that cystic follicles are a consequence of a mature follicle that fails to ovulate at the appointed time of ovulation in the oestrous cycle, accompanied by altered endocrine profiles [7,8]. These non-ovulating follicles may persist as the dominant follicle in the ovary for a long time, preventing the initiation of a new follicular wave and the further growth of large follicles, ultimately leading to ovulation defects and changes in the reproductive performance of livestock [9,10]. Despite the fact that extensive research has been conducted, the causes of cyst development and ovulation defects is still unknown.

As known energy sources from most foods, many studies have found that fatty acid metabolism in vivo is critical throughout the reproductive cycle of domestic animals [11]. Studies have found that the metabolic signatures of follicular fatty acid is a useful biological/diagnostic for identifying the pathophysiological mechanisms involved in the development of polycystic ovary syndrome (PCOS) [12,13]. In addition, studies have shown that the contents of fatty acids in follicles with different diameters were different. The percentage of stearate acid (SA) and polyunsaturated fatty acids (PUFAs) in follicular fluid gradually increased with increasing follicular diameter [14]. The increased levels of monounsaturated fatty acids (MUFAs) and PUFAs in follicles during winter may be responsible for the better reproductive performance than in summer [15]. Furthermore, studies have shown that the formation of porcine ovarian cysts was related to the disorder of fatty acid metabolism in follicular fluid [16]. Moreover, fatty acids are closely related to the reproductive potential of livestock.

Feed is the main source of fatty acids (FAs) for domestic animals; after digestion and absorption, fatty acids are induced to form lipid droplets (LDs) in cells [17]. During periods of under- and over-nutrition, the de-esterification of fatty acids from the stored lipids of the adipose tissue takes place by the action of a hormone-sensitive lipase, resulting in the temporary elevation of non-esterified fatty acids (NEFAs, also known as free fatty acids) in the circulation for coping with the body’s energy demands [18]. NEFAs are transported to the follicle through the blood–follicle barrier, and involved in cell proliferation, apoptosis, and steroid hormone synthesis, and other capabilities closely related to follicle development in recent years [11,19]. Studies have shown that saturated fatty acids, especially palmitic acid (PA) and stearic acid (SA), can induce the proliferation and apoptosis of granulosa cells and the maturation of oocytes in follicles [20,21,22]. However, other unsaturated fatty acids such as oleic acid and arachidonic acid (AA) play a positive role in follicular development but seem to depend on their concentration [23,24,25,26]. Therefore, the main purpose of this study was to reveal the changes in the fatty acid profile of porcine FF from pre-ovulatory, peri-ovulatory, and cystic follicles, and to hypothesize whether it is the alteration of fatty acids in the follicular fluid that ultimately leads to the success or failure of ovulation. It is helpful to further understand the regulation of fatty acids and their molecular mechanisms during ovulation, thereby providing a new direction for the precise feeding patterns of fatty acid supplementation throughout the reproductive cycle to improve the reproductive performance in domestic animals.

## 2. Materials and Methods

### 2.1. Follicular Fluid Isolation and Follicle Cell Collection

The study was approved by Guangxi Key Laboratory of Animal Breeding and Disease Control and in accordance with its guidelines for the care and use of experimental animals (GXU-2024-098). Porcine ovaries were collected from 5~6-month-old ternary hybrid pigs (Duroc × Landrace × Large white) at a local abattoir in Nanning (sampling time: March to August 2024; the average relative humidity was 79% and the mean temperature was 27 °C). The ovaries were placed in 4 °C sterile saline and transported to the laboratory within 2 h for further processing [16]. After washing with 75% ethanol, ovaries were placed on gauze, and then dissected to isolate individual follicles. According to the macroscopic characteristics [8,27], a single follicle that was pre-ovulatory (*n* = 6) and peri-ovulatory (*n* = 6) were isolated from normal ovaries, and cystic follicles (*n* = 6) were isolated from cystic ovaries. The outer ovarian tissue of follicles was separated as far as possible by ophthalmic tweezers, leaving a complete membrane enclosing the follicular fluid (FF). The FF was removed while carefully scraping down the mural granulosa cells with forceps, and the FF and granulosa complex was collected and centrifuged, and the remaining reddish follicular tissue were porcine theca cells (pTCs). After centrifugation (1200× *g*, 10 min), the upper FF (follicular fluid with a diameter of 7–10 mm is approximately 500–800 µL, while follicles with a diameter of about 2.5 cm have 5–6 mL) was used for subsequent hormone assay and metabolome, and the lower precipitate was pGCs. The pTCs and the pGCs were then collected and frozen in liquid nitrogen for subsequent RT-qPCR assay. Metabolomes of FF were studied from six cystic follicles samples, six pre-ovulatory follicles samples, and six peri-ovulatory follicles samples, each follicle came from a different sow.

### 2.2. Detection of Hormone Levels

Luteinizing hormone (LH), estradiol (E_2_), progesterone (P_4_), and follicle-stimulating hormone (FSH) in porcine FF were measured using Radioimmunoassay Kit (XIN FAN Biological Technology, China). According to the manufacturer’s instructions, add the components in separate tubes. Mix the components thoroughly and incubate the tubes or microplates for a specific period at an appropriate temperature, allowing the competitive binding between the labeled tracer and the analyte. After the incubation period, separate the precipitation (antibody–analyte complex) from the free fraction and measure the radioactivity in each precipitation using a gamma counter. Determine the concentration of the analyte in the tested samples by comparing their radioactivity measurements to the calibration curve. The sensitivities of Radioimmunoassay Kit were LH (≤1 mIU mL^−1^), E_2_ (≤2 pg mL^−1^), FSH (≤1 mIU mL^−1^), and P_4_ (≤1 ng mL^−1^). The percentage coefficients of variation (CV) are less than 10%.

### 2.3. Ovarian Tissues Section and Hematoxylin-Eosin (HE) Staining

Pre-ovulatory, peri-ovulatory, and cystic follicles were separated from ovaries. After being fixed with 4% paraformaldehyde (PFA) for 24 h, the follicles were embedded in paraffin, and then serially sliced into 5 μm sections, and every 5th slice was picked up for subsequent stained with HE. The tissue sections were deparaffinized with xylene, and rehydrated in a series of decreasing ethanol. Next, the sections were then stained with HE. Tissue section images were captured by the EVOS FL Auto microscope (Thermo Scientific,Waltham, MA, USA).

### 2.4. Transmission Electron Microscope (TEM) Staining

Follicular wall (1 × 1 × 1 cm^3^) separated from pre-ovulatory, peri-ovulatory, and cystic follicles were collected and washed twice with cold PBS, and fixed with 37 °C glutaraldehyde fixed solution (Servicebio, Wuhan, China) for 2–4 h, and transferred to 4 °C. The samples were washed 3 times with 0.1 M PBS, and then rehydrated in gradient decreasing ethanol and acetone for 15 min. Next, the samples were embedded in EMbed 812 (SPI, West Chester, PA, USA) and serially sliced into 80 nm by ultramicrotome (Leica UC7, Wetzlar, Germany). Sections were stained with uranyl acetate in pure ethanol for 15 min, and then rinsed with distilled water. Then, they were stained with lead citrate for 15 min and, again, rinsed with distilled water. The sections were air-dried overnight and we observed the organelles in granulosa cells and theca cells by TEM (Hitachi,Tokyo, Japan).

### 2.5. Real-Time Quantitative Polymerase Chain Reaction (RT-qPCR)

Total RNA form cells were extracted by VeZol Reagent (Vazyme, Nanjing, China) and quantified using a NanoDrop1000 Spectrophotometer (Thermo Scientific, Waltham, MA, USA) (OD260/OD280: 1.8–2.4). cDNA was synthesized by HiScript III RT SuperMix for qPCR (Vazyme, Nanjing, China) from 500 ng RNA. The qPCR program is shown in Table S1. qPCR was performed in a 20 μL reaction volume containing 2 × chamQ Universal SYBR qPCR Master Mix (Vazyme, Nanjing,China) by using LightCycler^®^ 96 instrument Real-Time PCR System (Roche, Basel, Switzerland). Analysis of the melting points of all samples was carried out to ensure amplification of correct products. The length of PCR products was checked after each run by agarose gel electrophoresis (3%, ethidium bromide stained). The 2^−ΔΔCt^ method was used to normalize and determine the RNA level relative to β-actin. All primers used are listed in Table S2.

### 2.6. Targeted Fatty Acid Metabolomics of Follicular Fluid

The metabolomics of fatty acid of FF was performed following the method of Hoving and Heijink [28]. Briefly, the first step is preparation of standard solutions where a mixed standard stock solution of 51 fatty acids (4000 µg mL^−1^) was diluted into ten points (1, 5, 10, 25, 50, 100, 250, 500, 1000, and 2000 µg mL^−1^). The second step is sample preparation that is the transmethylation of fatty acids from the total lipid fraction of FF. Ten microliters of each sample were then used to prepare quality control samples, which were used to determine the state of the chromatography–mass spectrometry system before injection, and also to evaluate the stability of the system during the experiment. Finally, GC-MS analysis was performed according to previously published protocols [28,29]. Sample preparation, mass spectrometry solvents, and GC-MS analysis were performed by Suzhou Panomix (Suzhou, China).

Methyl salicylate was obtained from Sigma-Aldrich (Shanghai, China). Mixed standard of 51 fatty acid methyl esters was obtained from NU-CHEK-PREP (Shanghai, China). The GC analysis was performed on trace 1300 gas chromatograph (Thermo Fisher Scientific,Waltham, MA, USA). The GC was fitted with a capillary column Thermo TG-FAME (50 m × 0.25 mm ID × 0.20 μm) and helium was used as the carrier gas at 0.63 mL/min. Injection was made in split mode at 8:1 with an injection volume of 1 μL and an injector temperature of 250 °C. The temperature of the ion source and transfer line were 300 °C and 280 °C, respectively. The column temperature was programmed to increase from an initial temperature of 80 °C, which was maintained for 1 min, followed by an increase to 160 °C at 20 °C/min, which was maintained for 1.5 min, and increase to 196 °C at 3 °C/min, which was maintained for 8.5 min, and, finally, an increase to 250 °C at 20 °C/min, and this temperature was maintained for 3 min. Mass spectrometric detection of metabolites was performed on ISQ 7000 (Thermo Fisher Scientific, USA) with electron impact ionization mode. Single-ion monitoring (SIM) mode was used with the electron energy of 70 eV. Calculation formula was as follows: the content of sample (µg mL^−1^) = C (µg mL^−1^) × 1/Amount (μL) × 1000.

Principal component analysis (PCA) and orthogonal partial least squares–discriminant analysis (OPLS-DA) models were constructed from the metabolomics data. R2Y and Q2Y were used to evaluate the validity of the model. The variable importance in the projection (VIP) of the first principal component obtained from the OPLS-DA analysis was determined. The metabolomics data and screening of differential fatty acids were statistical analyzed on the cloud platform of Suzhou Panomix (https://www.biodeep.com (accessed on 26 January 2025)).

### 2.7. Statistical Analysis

All data were presented as means ± SEM based on at least three independent experiments. All data were normally distributed by the Shapiro–Wilk normality test. The difference between different groups of qPCR results was analyzed by Student’s t test while hormone concentration and fatty acids content results were analyzed by one-way ANOVA with Tukey’s multiple-comparison test. All statistical analyses were performed using GraphPad Prism version 8.0 (GraphPad Software, Boston, MA, USA) and *p* values ≤ 0.05 were considered statistically significant.

## 3. Results

### 3.1. Classification of Pre-Ovulatory, Peri-Ovulatory, and Cystic Follicles in Sows

**Figure 1:**
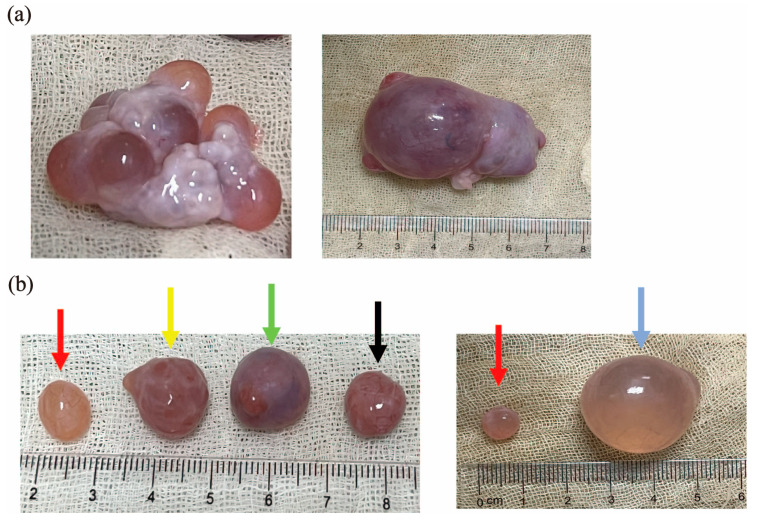
The macroscopic characteristic of pre-ovulatory and peri-ovulatory follicles from normal ovulation ovary and cystic follicles from cystic ovaries: (**a**) normal ovulation ovary (left) and cystic ovaries (right); and (**b**) pre-ovulatory follicle (red arrows), peri-ovulatory follicle (yellow arrows), corpus hemorrhagicum (green arrows), and corpus luteum (black arrows) from normal ovulation ovary; pre-ovulatory follicle (red arrows) from normal ovulation ovary; and cystic follicle (blue arrows) from cystic ovaries.

In order to explore the characteristics of follicles in different ovulation stages with a diameter of >7 mm and the mechanism of ovulation defects in cystic follicles, we collected pre-ovulatory and peri-ovulatory follicles from normal ovaries and cystic follicles from cystic ovaries to analyze the differences in the macroscopic characteristics of the above three follicles. According to the macroscopic characteristics [8,27], we found pre-ovulatory follicles prior to the LH surge and peri-ovulatory follicles following LH stimulation immediately prior to ovulation, while cystic follicles have ovulation disorders with significantly increased diameters. As shown in Figure 1, there were significant differences in macroscopic characteristics between the pre-ovulatory, peri-ovulatory, and cystic follicles of pigs. Compared with the peri-ovulatory follicles, corpus hemorrhagicum, and corpus luteum (Figure 1a), the walls of pre-ovulatory follicles were thinner, fuller and more flexible. Abundant capillaries were observed on the surface of the pre-ovulatory follicles and the follicular fluid was luminous yellow, while red, thick follicular walls and an obvious ovulation rupture site were observed in peri-ovulatory follicles. Cystic follicles are similar in their macroscopic characteristics to pre-ovulation follicles, but can be up to 25 mm in diameter (Figure 1b).

### 3.2. Biological Characteristics of Pre-Ovulatory, Peri-Ovulatory, and Cystic Follicles Include Histology, Hormonal Assays, and Molecular Biology

**Figure 2:**
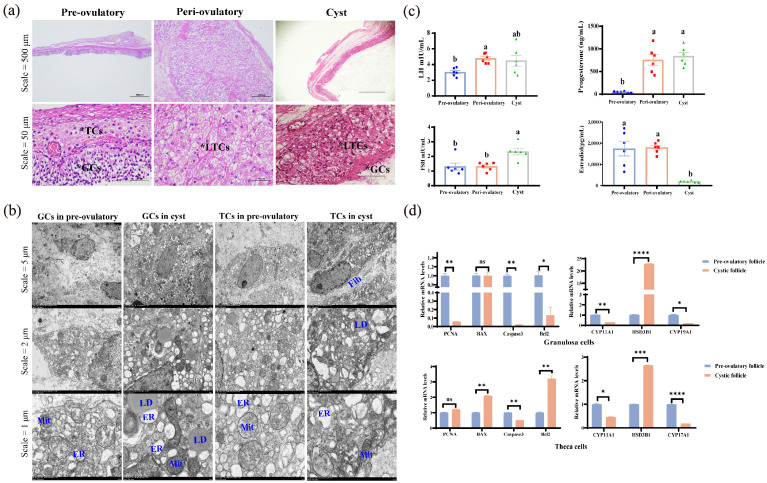
Histologic, hormonal, and molecular findings of pre-ovulatory, peri-ovulatory, and cystic follicles: (**a**) HE-stained sections of pre-ovulatory, peri-ovulatory, and cystic follicles (*GCs: granulosa cells; *TCs: theca cells; and *LTCs: luteinized theca cells); (**b**) the subcellular structure of GC and TC in pre-ovulatory and cystic follicle observed by TEM (Note: Mit for mitochondria, ER for endoplasmic reticulum, LD for lipid droplets, and Fib for fibrosis); (**c**) hormone levels in follicular fluid of pre-ovulatory, peri-ovulatory, and cystic follicles (*n* = 6); and (**d**) detection of GC- and TC-function-related gene expression in pre-ovulatory and cystic follicle of pig (*n* = 3). a, b values with different superscript letters within column are significantly different (*p* ≤ 0.05), and same superscript letters are not significantly different (*p* > 0.05); * *p* ≤ 0.05, ** *p* ≤ 0.01, *** *p* ≤ 0.0005, and **** *p* ≤ 0.0001, ns (no significance).

HE staining showed that the theca cell layer and granulosa cell layer in peri-ovulatory follicles began to carry out luteinization compared to pre-ovulatory follicles, but cystic follicles showed the luteinization of theca cells layer, as well as the loss and shedding of the granulosa cell layer (Figure 2a).

Transmission electron microscope observations showed that the presence of LDs can be observed in TCs and GCs in both pre-ovulatory and cystic follicles. The ultrastructure of cystic follicles mainly showed severe endoplasmic reticulum (ER) stress in granulosa cell and fibrosis of theca cells compared to the pre-ovulatory follicle (Figure 2b).

Subsequently, the LH, E_2_, P_4_, and FSH levels in the FF of pre-ovulatory, peri-ovulatory, and cystic follicles were assayed (Figure 2c). Pre-ovulatory follicles were characterized by high levels of estrogen and low levels of progesterone and LH (*p* ≤ 0.05), while peri-ovulatory follicles have elevated LH levels, accompanied by increased levels of estrogen and progesterone (*p* ≤ 0.05). Different from the normal ovulation process, cystic follicles showed an abnormal decrease in estrogen (*p* ≤ 0.05), accompanied by an increase in LH, progesterone, and FSH (*p* ≤ 0.05).

The RT-qPCR results showed that the expression levels of genes related to the proliferation and apoptosis of granulosa cells and theca cells in cystic follicles were disordered, with weak proliferation activity and low apoptosis frequency (Figure 2d). Meanwhile, the expression levels of *CYP11A1*, *CYP17A1,* and *CYP19A1* were significantly decreased in the GCs or TCs of cystic follicles (*p* ≤ 0.05), respectively, while *HSD3B1* was significantly increased both in the GCs and TCs of cystic follicles (*p* ≤ 0.05) (Figure 2d).

### 3.3. The Profile of Fatty Acid in FF from Pre-Ovulatory, Peri-Ovulatory, and Cystic Follicles

**Figure 3:**
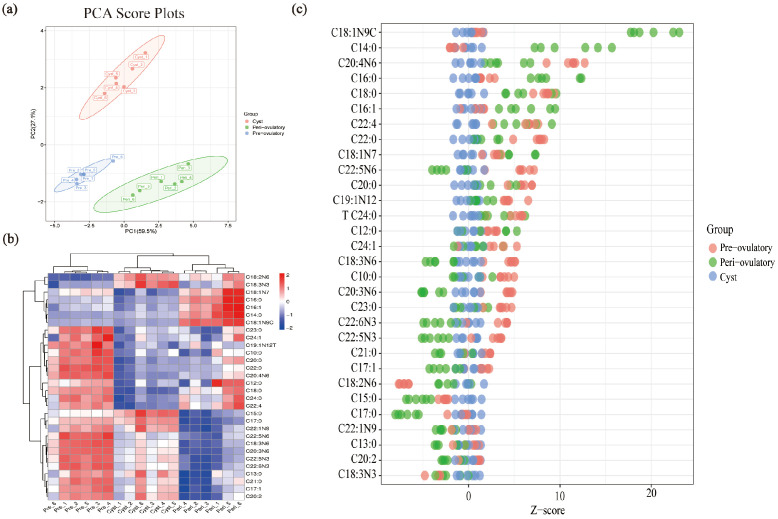
Overview of fatty acid metabolomics of FF from pre-ovulatory, peri-ovulatory, and cystic follicles (*n* = 6): (**a**) PCA scores plot of all samples; (**b**) clustering heat map of all samples; and (**c**) Z-score of FAs across the three groups.

Targeted fatty acid metabolome was used to detect the contents of fifty-one fatty acids in the follicular fluid of pre-ovulatory, peri-ovulatory, and cystic follicles; the fatty acid contents of each sample are listed in Table S3. A principal component analysis (PCA) showed differences in the metabolic profiles of different samples (Figure 3a). The heat map (Figure 3b) and Z-score maps (Figure 3c) further show the changes in fatty acid content in these three follicles. The results show that the Top 5 abundant fatty acids in the FF of pre-ovulatory, peri-ovulatory, and cystic follicles were linoleic acid (LA, C18:2N6), oleate acid (OA, C18:1N9C), palmitate acid (PA, C16:0), stearate acid (SA, C18:0), and arachidonic acid (AA, C20:4N6), while the category of PUFAs were the highest-proportion FAs in the FF of the three groups (Table 1). LA (77.31 ± 0.495 µg mL^−1^) was the most abundant fatty acid in the FF of pre-ovulatory follicles, while OA (105.32 ± 2.12 µg mL^−1^) was the most abundant fatty acid in the FF of peri-ovulatory follicles. LA (95.91 ± 1.07 µg mL^−1^) is also the most abundant fatty acid in the FF of cystic follicles, but its content is much higher than that of other FAs in the FF of cystic follicles (Table 1). The content of OA (59.25 ± 0.94 µg mL^−1^), SA (39.12 ± 0.69 µg mL^−1^) and AA (25.02 ± 0.65 µg mL^−1^) in the FF of cystic follicles was significantly decreased among the three groups (*p* ≤ 0.05).

### 3.4. Analysis of Target Fatty Acid Metabolomics in FF from Pre-Ovulatory and Cystic Follicles

**Figure 4:**
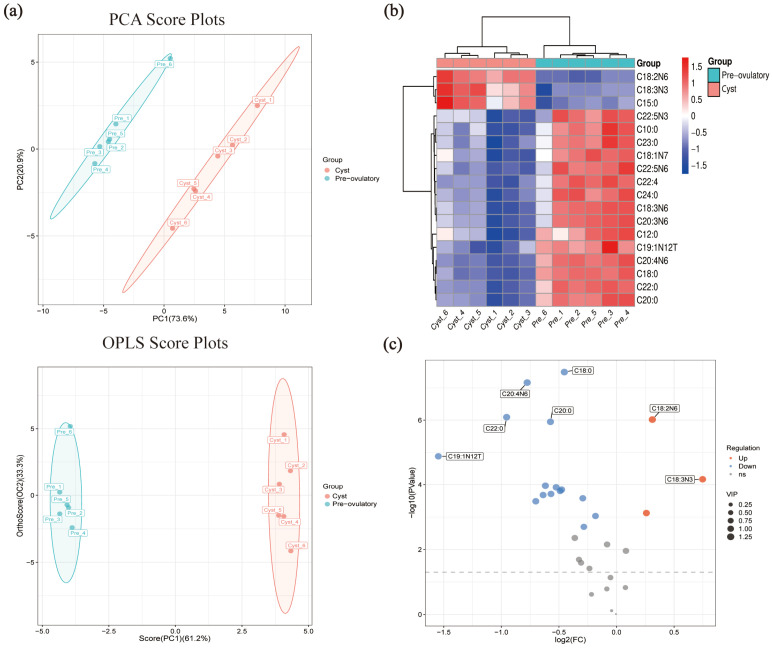
Differential fatty acid identification of FF between pre-ovulatory and cystic follicles (*n* = 6): PCA and OPLS scores plot (**a**), clustering heat map (**b**), and the Volcano plot diagrams (**c**). Dotted lines mean: −log10(*p* value) = −log10(0.05) = 1.3.

In order to screen the differential fatty acids (DEFAs) associated with ovulation defects in cystic follicles, the DEFAs of pre-ovulation and cystic follicles were further analyzed. The PCA model (R2X(cum) 0.945) and OPLS-DA model (R2X(cum) 0.945, Q2Y 0.994) were clearly discriminated between cystic and pre-ovulatory follicles (Figure 4a).

A total of 18 DEFAs (*p*-value *≤* 0.05 and VIP ≥ 1) were identified in the FF between pre-ovulatory and cystic follicles (Table 2); we performed a heat map (Figure 4b) and volcano map (Figure 4c) analysis based on the DEFAs. The results showed that LA (95.91 ± 1.07 µg mL^−1^), alpha-LA (1.29 ± 0.06 µg mL^−1^), and pentadecanoate acid (0.72 ± 0.02 µg mL^−1^) were the only three DEFAs that were significantly increased in the FF of cystic follicles (*p* ≤ 0.05) (Table 2 and Table S3). The contents of AA (25.02 ± 0.65 µg mL^−1^), SA (39.12 ± 0.69 µg mL^−1^), and gamma-LA (1.23 ± 0.05 µg mL^−1^) in the FF of cystic follicles were significantly reduced (*p* ≤ 0.05) (Table 2 and Table S3).

**Figure 5:**
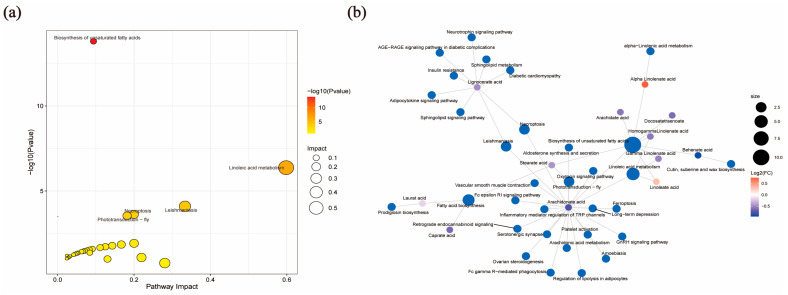
KEGG annotation of differential fatty acids in the FF of pre-ovulatory and cystic follicles: (**a**) KEGG pathway enrichment scatter plot; and (**b**) KEGG pathway enrichment network diagram.

Metabo Analyst 5.0 software was used to investigate DEFAs biological functions and build pathways by KEGG pathway analysis. We showed the Top 5 enrichment KEGG pathways of DEFAs in the FF of pre-ovulatory and cystic follicles (Table 3), and the DEFAs were significantly enriched via the biosynthesis of unsaturated fatty acids and linoleic acid metabolism (Figure 5a). The pathway with the greatest impact value is the linoleic acid metabolic pathway, and LA and AA on this pathway are the DEFAs with the most significant differences and the most enriched metabolites (Figure 5b).

## 4. Discussion

Successful ovulation is a key capability of reproductive performance in livestock [30]. First, we collected pre-ovulatory and peri-ovulatory follicles at different stages of normal ovulation as well as cystic follicles with ovulation defects for characterization. One of the typical characteristics of cystic follicles is an increase in diameter, which is the underlying diagnostic criterion for determining cystic follicles in many animals [8]. The results of this study showed that the pre-ovulatory follicles, peri-ovulatory follicles, corpus hemorrhagicum, and corpus luteum in normal porcine ovaries are between 7–15 mm in diameter, whereas cystic follicles can reach up to 25 mm in diameter, which can be easily differentiated from other follicles by their diameter (Figure 1).

In addition, the shedding and loss of the granulosa cell layer and the decrease in GC proliferation and apoptosis were observed in the cystic follicles of mice [31], cattle [32], and pigs [33,34], and the same phenomenon was observed in this study, which showed exfoliated and less layered GC in the histological structures of cystic follicles (Figure 2a). The quantitative PCR results further verified that *PCNA*, *Caspase3,* and *Bcl2* were significantly decreased in the GCs of cystic follicles, but showed significant increases in *BAX* and *Bcl2* and a significant decrease in *Caspase3* in the TCs of cystic follicle (Figure 2d). The proliferation and apoptosis of GCs and TCs in the cystic follicles are in a balanced state, resulting in a slow growth of the cystic follicle without degeneration, allowing for the prolonged presence of cystic follicles in the ovary.

Transmission electron microscopy showed that a large number of lipid droplets which are substrates for steroid synthesis were observed in pre-ovulatory and cystic follicles (Figure 2b), indicating that follicles during ovulation are in the active phase of steroid synthesis. In the present study, the LH, E_2_, P_4_, and FSH concentrations in the FF of pre-ovulatory, peri-ovulatory, and cystic follicles were assayed (Figure 2c). The E_2_ in the cystic follicle was much lower than in pre-ovulatory follicles, while P_4_ in the cystic follicle was comparable to that in the peri-ovulatory follicles, whereas the levels of LH in the cystic follicle were between the pre-ovulatory and peri-ovulatory follicles. This hormone assay is consistent with the study by Sun et al. [34]. Interestingly, FSH was the highest in cystic follicles, while FSH is the hormone associated with an increased follicle diameter [35]. It is widely known that *CYP17A1,* which was expressed in TCs, is responsible for the synthesis of androstenedione, and then the transfer to GCs, which act as the location of E_2_ synthesis in the follicle, converting androstenedione to estrogen under the action of CYP19A1 [36]. In cystic follicles, not only were the loss and shedding of GCs observed (Figure 2a), but, also, the expression of *CYP17A1* in TCs and *CYP19A1* in GCs were significantly decreased (Figure 2d). Ultimately, the estrogen level in the FF of cystic follicles is much lower than that in the pre-ovulatory follicles, and the rapid increase in estrogen is a signal that the follicle begins to ovulate [37]. In addition, the expression of *HSD3B1*, which is responsible for the important regulatory factor of progesterone synthesis [38], was significantly increased in both the GCs and TCs of cystic follicles (Figure 2d). At the same time, the TCs in cystic follicles began to luteinized (Figure 2a), which is the main reason why the P_4_ in cystic follicles was higher than in pre-ovulatory follicles reported in sows [34,39]. In this study, porcine cystic follicles showed a significant decrease in E_2_ and a significant increase in P_4_ compared to pre-ovulatory follicles. Because of this abnormal change, the low amounts of E_2_ in the cystic follicles cannot reach the threshold necessary to trigger the pre-ovulatory LH surge. Meanwhile, the abnormally increased level of P_4_ exerts negative feedback on the hypothalamo–hypophyseal axis which further inhibited the LH surge. The tonic center continues to pulse under the conditions of low E_2_ and high P_4_, although at a slower rate, allowing for a sustained and pulsatile release of pituitary LH, which promotes the growth of the cystic follicles—a structure that does not regress within the timeframe typical of a normal follicle.

We performed targeted fatty acid metabolomics on the FF from pre-ovulatory, peri-ovulatory, and cystic follicles in porcine for the first time. The results showed that LA, OA, PA, SA, and AA were the top five FAs in the FF of pre-ovulatory, peri-ovulatory, and cystic follicles in this study which was consistent with that reported in the FF of pigs, cattle, and sheep [11]. Furthermore, these five FAs are significantly different in health/atresia follicles from buffalo and pigs [40,41], suggesting that these five FAs play a critical role in the development and maturation of follicles, and even during the ovulation process [18]. Notably, the results showed that the total amount of FAs in the FF of peri-ovulatory follicles was the highest (Table S3), which indicates that a large number of FAs were transported and transformed in blood vessels and follicles during ovulation [19,42]. This result suggests that an increased demand for fatty acids in sows during the ovulation. We found the percentage of PUFAs in the three types of follicles were both the highest (Table 1), suggesting that increased PUFAs play a crucial role in the ovulation and rupture of follicles. Similar to the findings of YAO et al. [14], they also found that, with the increase in the diameter of porcine follicles, the content of PUFAs gradually increased.

The enrichment analysis of KEGG metabolic pathways showed that DEFAs were significantly enriched via the biosynthesis of unsaturated fatty acids and the linoleic acid metabolism, which is an essential fatty acid that can only be obtained through diet [11]. It has been shown that LA is synthesized into alpha-LA via ω-3 fatty acid desaturase, whereas gamma-LA and AA are synthesized via ω-6 fatty acid desaturase [43]. Notably, LA and alpha-LA were significantly increased in the FF of cystic follicles, whereas gamma-LA and AA were significantly decreased in the FF of cystic follicles (Table 2). This result implies that the ω-6 fatty acid desaturase, a key enzyme in the biosynthesis pathway of LA, suggests a possible reduction in ovulation-deficient sows. Our research indicates that LA and its downstream metabolites play an important role in the follicular ovulation stage, implying that appropriate changes in the proportion of LA, alpha-LA, gamma-LA, and AA to the feed may improve the reproductive performance of sows.

It is well-known that AA is the direct precursor of bioactive lipid metabolites of eicosanoids such as prostaglandins, leukotrienes, and epoxyeicosatrienoic acid [44], and the PGs (especially PGE2 and PGF2α) play a crucial role in ovulation and the formation of the corpus luteum [27]. In the present study, we detected a significant decrease in AA in the FF of porcine cystic follicles (Table 1), which is likely to be the cause of the ovulation defects of follicles and the eventual formation of cysts. In addition, this study also found that SA and lauric acid (C12:0) were also DEFAs screened from pre-ovulatory and cystic follicles. And the same results were also found in the FF of PCOS [45] and in the GCs and TCs of porcine cystic follicles [16,46], indicating that SA and lauric acid were also involved in the formation of cystic follicles.

We speculate that the alteration of the nutritional level of sows has caused changes in the fatty acid metabolism in the follicular microenvironment, ultimately leading to the success or failure of ovulation. In addition, the limitations of this study were the low sample number of follicles, and the fact that all three categories of follicles (such as the signatures of fatty acids in the follicular fluid between pre-ovulatory and peri-ovulatory follicles) also need to be further analyzed. Furthermore, as a systemic metabolic disease, the pathogenesis of ovarian cysts may be further clarified by combining the changes in fatty acids in the blood and follicular fluid.

## 5. Conclusions

For the first time, our study explored the biochemical characterization and fatty acid metabolic signatures of follicular fluid in pre-ovulatory, peri-ovulatory, and cystic follicles in sows, highlighting that ovulation defects in cystic follicles are very likely related to the significantly increased LA and significantly decreased AA in the follicular fluid, and the reason for this change may be related to a possible reduction in the ω-6 desaturase activity of the linoleic acid metabolism pathway. These findings indicate a strong correlation between LA, AA, and ovulation defects in cystic follicles, and the underlying mechanisms of the association between LA, AA, and ovulation needs to be further explored.

## Figures and Tables

**Table 1:** The content and classification of fatty acids detected in pig follicles.

Content (µg mL^−1^)	LA C18:2N6		OA C18:1N9C	PA C16:0		SA C18:0		AA C20:4N6
Pre-ovulatory	77.31 ± 0.495 ^c^		61.45 ± 0.47 ^b^	60.54 ± 0.54 ^b^		53.55 ± 0.54 ^a^		42.83 ± 0.92 ^a^
Peri-ovulatory	91.41 ± 1.66 ^b^		105.32 ± 2.12 ^a^	75.02 ± 1.89 ^a^		50.05 ± 1.70 ^a^		31.21 ± 1.34 ^b^
Cyst	95.91 ± 1.07 ^a^		59.25 ± 0.94 ^c^	57.19 ± 0.79 ^b^		39.12 ± 0.69 ^b^		25.02 ± 0.65 ^c^
**Composition**		**SFAs**			**MUFAs**		**PUFAs**	
Pre-ovulatory		35.9%			22.4%		41.7%	
Peri-ovulatory		34.1%			30.7%		35.2%	
Cyst		33.1%			23%		43.9%	

^a,b,c^ Values with different superscript letters within column are significantly different (*p* ≤ 0.05), and same superscript letters are not significantly different (*p* > 0.05). The values are the means ± SEMs of six individuals.

**Table 2:** Differential FAs between pre-ovulatory and cystic follicle.

Abbreviation	Name	VIP Value	Fold Change	log2 (FC_cyst/pre)	*p* Value
C18:2N6	Linoleic	1.257	1.240	0.31	9.6 × 10^7^
C20:4N6	Arachidonic	1.252	0.584	−0.78	6.8 × 10^8^
C18:0	Stearate	1.250	0.730	−0.45	3.2 × 10^8^
C22:0	Behenate	1.223	0.516	−0.95	8.1 × 10^7^
C20:0	Arachidate	1.217	0.672	−0.57	1.1 × 10^6^
C22:4	Docosatetraenoate	1.180	0.652	−0.62	1.1 × 10^4^
C18:3N3	Alpha Linolenate	1.156	1.679	0.75	6.8 × 10^5^
C24:0	Lignocerate	1.148	0.719	−0.48	1.4 ×10^4^
C15:0	Pentadecanoate	1.146	1.197	0.26	7.5 × 10^4^
C22:5N6	DPA	1.144	0.615	−0.70	3.3 × 10^4^
C18:3N6	Gamma Linolenate	1.139	0.695	−0.52	1.2 × 10^4^
C10:0	Caprate	1.118	0.643	−0.64	2.1 × 10^4^
C20:3N6	HomogammaLinolenate	1.117	0.674	−0.57	1.9 × 10^4^
C23:0	Tricosanoate	1.116	0.712	−0.49	1.6 × 10^4^
C19:1N12T	7-Transnonadecenoate	1.115	0.342	−1.55	1.3 × 10^5^
C18:1N7	Vaccenate	1.105	0.816	−0.29	2.6 × 10^4^
C12:0	Laurate	1.060	0.881	−0.18	9.3 × 10^4^
C22:5N3	DPA	1.019	0.821	−0.28	2.0 × 10^3^

Note: Fold change is the ratio of the cystic follicle to the pre-ovulatory follicle, FC > 1 means up-regulated, and FC < 1 means down-regulated.

**Table 3:** Top 5 enrichment KEGG pathways of DEFAs in the FF of pre-ovulatory and cystic follicles.

KEGG Metabolic Pathway	*p* Value	Impact
Biosynthesis of unsaturated fatty acids	<0.0001	0.0938
Linoleic acid metabolism	<0.0001	0.5982
Leishmaniasis	<0.0001	0.3333
Necroptosis	0.0001	0.2
Fatty acid biosynthesis	0.0002	0.0006

## Data Availability

The authors confirm that the data supporting the findings of this study are available within the article and its Supplementary Materials.

## Supplementary Materials

The following supporting information can be downloaded at: https://www.mdpi.com/article/10.3390/metabo15070421/s1, Table S1: The qRT-PCR program in this study; Table S2: The primers used for the qRT-PCR in this study; Table S3: Fatty acid content of follicular fluid from porcine pre-ovulatory, peri-ovulatory, and cystic follicles.

## Abbreviations

The following abbreviations are used in this manuscript:

BAX	Bcl-2-associated x-protein
Bcl2	B-cell lymphoma/leukemia 2
Caspase3	Cysteine-aspartic acid protease 3
CYP11A1	Cytochrome P450 Family 11 Subfamily A Member 1
CYP17A1	Cytochrome P450 Family 17 Subfamily A Member 1
CYP19A1	Cytochrome P450 Family 19 Subfamily A Member 1
PCNA	Proliferating cell nuclear antigen
PGs	Prostaglandins
PGE2	Prostaglandin E2
PGF2α	Prostaglandin F2α
