# Supplementary material for: Follicular Biochemical Characterization and Fatty Acid Metabolic Signatures of Follicles During Ovulation Process Reveal the Potential Mechanism for Ovarian Cyst Formation in Sows
Source: Metabolites. 2025 Jun 20;15(7):421. doi: 10.3390/metabo15070421 (PMC12298436; doi:10.3390/metabo15070421)
Supplement: Supplementary file 1 [file metabolites-15-00421-s001.zip › metabolites-3682432-supplementary-Tables S1 and S2.pdf]

Table S1. The qRT-PCR program in this study.

**Stage 1**	Hot-Start DNA Polymerase Activation	Reps: 1	95 ℃	30 sec
**Stage 2**	PCR	Reps: 40	95 ℃<br>60 ℃	10 sec<br>30 sec
**Stage 3**	Melt Curve	Reps: 1	95 ℃<br>60 ℃<br>95 ℃	15 sec<br>60 sec<br>15 sec

Table S2. The primers used for the qRT-PCR in this study.

Genes	Primers (5′-3′)	Amplicon lengths (bp)
β-actin	F: GATGACGATATTGCTGCGCT	248
	R: TTCTCCATGTCGTCCCAGTT	
PCNA	F: CCTGAAGAAGGTGCTGGAAG	269
	R: GTGCCAAGGTGTCTGCATTA	
BAX	F: AAGCGCATTGGAGATGAACT	159
	R: AAAGTAGAAAAGCGCGACCA	
Caspase3	F: TTGGACTGTGGGATTGAGACG	165
	R: CGCTGCACAAAGTGACTGGA	
Bcl-2	F: ATGTGTGTGGAGAGCGTCAA	187
	R: CCTTCAGAGACAGCCAGGAG	
CYP11A1	F: TGCATCTCCACTAAAACCCC	288
	R: CTGGTAATGCTGGTGATAGG	
HSD3B1	F: TCCACACCAGCAGCATAGAG	245
	R: CATGTGGGCAAAGATGAATG	
CYP19A1	F: GCTAATTGCAGCACCAGACA	195
	R: GGCTGGTACCTCATGCTCTC	
